# Impact of Metallic Nanoparticles on In Vitro Culture, Phenolic Profile and Biological Activity of Two Mediterranean Lamiaceae Species: *Lavandula viridis* L’Hér and *Thymus lotocephalus* G. López and R. Morales

**DOI:** 10.3390/molecules26216427

**Published:** 2021-10-25

**Authors:** Sandra Gonçalves, Inês Mansinhos, Raquel Rodríguez-Solana, Gema Pereira-Caro, José Manuel Moreno-Rojas, Anabela Romano

**Affiliations:** 1 MED-Mediterranean Institute for Agriculture, Environment and Development, Faculdade de Ciências e Tecnologia, Universidade do Algarve, Campus de Gambelas, 8005-139 Faro, Portugal; ifmansinhos@ualg.pt (I.M.); or raquel.rodriguez.solana@juntadeandalucia.es (R.R.-S.); 2 Department of Food Science and Health, Andalusian Institute of Agricultural and Fisheries Research and Training (IFAPA), Avenida Menendez-Pidal, SN, 14004 Córdoba, Spain; mariag.pereira@juntadeandalucia.es (G.P.-C.); josem.moreno.rojas@juntadeandalucia.es (J.M.M.-R.)

**Keywords:** antioxidant activity, enzymes inhibition, in vitro culture, nanoparticles, phenolics, rosmarinic acid

## Abstract

Nanoparticles (NPs) recently emerged as new chemical elicitors acting as signaling agents affecting several processes in plant metabolism. The aim of this work was to investigate the impact of the addition of copper oxide (CuO), zinc oxide (ZnO) and iron oxide (Fe_3_O_4_) NPs (<100 nm) at different concentrations (1, 5 and 10 mg/L) to the culture media on several morphological, physiological and -biochemical parameters of in vitro shoot cultures of *Lavandula viridis* L’Hér and *Thymus lotocephalus* G. López and R. Morales (Lamiaceae), as well as on phenolic profile and bioactivity (antioxidant and enzyme inhibition capacities). Although some decreases in shoot number and length were observed in response to NPs, biomass production was not affected or was improved in both species. Most NPs treatments decreased total chlorophyll and carotenoid contents and increased malondialdehyde levels, an indicator of lipid peroxidation, in both species. HPLC-HR-MS analysis led to the identification of thirteen and twelve phenolic compounds, respectively, in *L. viridis* and *T. lotocephalus* extracts, being rosmarinic acid the major compound found in all the extracts. ZnO and Fe_3_O_4_ NPs induced an increase in total phenolic and rosmarinic acid contents in *T. lotocephalus* extracts. Additionally, some NPs treatments also increased antioxidant activity in extracts from this species and the opposite was observed for *L. viridis*. The capacity of the extracts to inhibit tyrosinase, acetylcholinesterase and butyrylcholinesterase enzymes was not considerably affected. Overall, NPs had a significant impact on different parameters of *L. viridis* and *T. lotocephalus* in vitro shoot cultures, although the results varied with the species and NPs type.

## 1. Introduction

Natural resources contain a great diversity of chemical molecules produced as a response to several biotic and abiotic factors affecting organisms’ biosynthetic pathways. Among natural sources, plants have a special relevance due to their great diversity of molecules with beneficial biological activities important to humans and their single structural features, namely safety, multi-target spectrum and metabolite likeness [1,2]. Lamiaceae is a family of plant species with wide distribution in different ecosystems comprising species with great economic value, due to their potential as ornamentals, culinary herbs and due to the production of valuable bioactive compounds [3,4]. *Lavandula* and *Thymus* are two of the most important genera within Lamiaceae family, comprising species producing high-value phytochemicals as volatiles (essential oils) and phenolics [5,6,7]. *Lavandula viridis* L’Hér and *Thymus lotocephalus* G. Lopez and R. Morales are Mediterranean aromatic species endemic to the south-west Iberian Peninsula and the Algarve region (South of Portugal), respectively. Both species produce bioactive phytochemicals with antioxidant and enzyme inhibiting properties [8,9,10,11,12,13,14].

Lately, more attention is dedicated to in vitro plant tissue culture systems as means for bioactive compounds production [2,15] including from *Lavandula* and *Thymus* species [14,16]. In vitro culture techniques allow the rapid propagation of disease-free plants, genetic transformation of plants, and also the rapid production of notable secondary metabolites [15]. The production of plant-derived metabolites in vitro presents some advantages in comparison with traditional systems, such as the rapid production, low burden of human pathogens and scalability, and the production throughout the year with no seasonal restrictions [2,15,17]. This method allows the mass production of plant material without negative impact on natural populations and, thus is particularly important for species that are threatened or at the risk of extinction as is the case of *T. lotocephalus*. Many reports suggest that plant natural habitats are being rapidly lost due to many factors including urbanism, climate change and agriculture. Plant biotechnology using tissue culture techniques may ensure the ex situ conservation of those plants and concurrently provide a sustainable method for the production of valuable bioactives [2].

Nanotechnology, which involves the use of many nanomaterials with dimensions in the range from 1 to 100 nm (nanoparticles, nanocoatings, nanofilms, etc.), has currently swarmed over all areas of life, having enormous applications in the fields of food, cosmetics, and medicine. Among nanomaterials, nanoparticles (NPs) have unique properties possessing specific physico-chemical properties attributed to their smaller size, large surface area and high reactivity compared to their bulk counterparts [18]. Recent studies showed also encouraging inputs from nanotechnology in plant biotechnology including plant tissue culture [19]. The application of NPs has been successfully reported in plant regeneration, callus induction, genetic transformation, elimination of microbial contaminants, and particularly on secondary metabolite production [19]. NPs emerged as new chemical elicitors acting as signalling agents affecting several morphological, physiological and biochemical processes in plant metabolism [19,20]. Elicited in vitro plant cultures are recently gaining more interest for their potential in the production of high-value secondary metabolites. Various biological, chemical and physical elicitors can trigger a signal transduction cascade of defense systems in plants mediating the expression of genes linked to the biosynthesis of secondary metabolites [21]. Despite the insights achieved through some previous studies on in vitro culture of *L. viridis* and *T. lotocephalus* [22,23] and the feasibility of in vitro-produced shoots for the production of secondary metabolites [11,12,14], to the best of our knowledge, there are no previous reports on the effects of NPs on in vitro culture of these species nor in their potential impact on elicitation of secondary metabolites with biological properties. As NPs can act as chemical elicitors and copper, zinc and iron are microelements essential for plant growth and development, the present study aimed to elucidate if copper oxide (CuO), zinc oxide (ZnO) and iron oxide (Fe_3_O_4_) NPs (<100 nm), at different concentrations (1, 5 and 10 mg/L), can affect morphological, physiological and biochemical features of in vitro shoots of these species, as well as the production of phenolic compounds, antioxidant and enzyme inhibitory activities.

## 2. Results and Discussion

### 2.1. Biometric and Physiological Features

Several biotic and abiotic elicitors acting as stress agents can affect the growth of in vitro cultures. Both stimulatory and inhibitory effects of the addition of different metal oxide (e.g., CuO, ZnO and NiO) NPs to the culture medium on shoot growth have been reported [24,25,26]. As frequently reported, in this work the effect of the addition of NPs to the culture medium depended on the type of NP tested and the plant species. In *L. viridis* the number of shoots produced was not significantly affected by Fe_3_O_4_ NPs at any dose used (average of 4.26 in the control and 3.92–4.41 in Fe_3_O_4_ NPs) but was negatively affected by CuO (from 2.84 to 3.46) and ZnO (2.33–3.07) NPs in a concentration-dependent manner (Table 1). Although the three NPs tested negatively affected shoot length in *L. viridis*, the biomass produced (fresh and dry mass) in NPs-treated cultures was similar or even higher than the obtained with the control medium (Table 1). In *T. lotocephalus*, ZnO NPs did not affect any growth parameter, and Fe_3_O_4_ NPs significantly improved shoot number at 5 and 10 mg/L (16.28, 31.62 and 26.35 in the control, NPs at 5 and 10 mg/L, respectively) and biomass production at 5 mg/L (fresh weight: 1902.07 mg in the control and 4854.29 g in Fe_3_O_4_ NPs; dry weight: 174.43 and 343.31, respectively). On the other hand, CuO NPs significantly reduced shoot number (from 16.28 in the control to 8.19–11.19 in CuO NPs) and length (from 38.30 mm to 17.54–29.44 mm) but did not affect biomass production (Table 1). In line with these results, the growth inhibitory effect of several ZnO and CuO NPs on different types of in vitro cultures has been reported by different authors. ZnO NPs reduced the growth of *Solanum melongena* seedlings [27]. High concentrations of CuO and ZnO NPs also reduced shoot and root growth of in vitro grown seedlings of *Abelmoschus esculentus* (okra) [24]. High concentrations of CuO NPs reduced shoot and root growth in *Oryza sativa* plants [28]. Several investigations demonstrated that the effects of NPs on growth are very dependent on the concentration used. For instance, Javed et al. [29] reported a positive role of ZnO NPs in the growth of *Stevia rebaudiana* shoots up to 1 mg/L but higher concentrations caused a significant reduction in growth parameters. In this work, overall ZnO and CuO NPs harmed the shoot multiplication at all concentrations tested however did not significantly affect biomass production. On the other hand, Fe_3_O_4_ NPs slightly improved shoot growth (Table 1). It is widely accepted that mineral composition of the culture medium influences parameters such as cellular differentiation, phenotypic characteristics, plant growth and development in in vitro conditions and that controlling micronutrients levels is more important in in vitro conditions than in vivo [30]. In this sense, despite being a micronutrient, iron demonstrated a great influence on in vitro plant growth and, when applied in the form of nanoparticles, the smaller the diameter of iron the easiest it is absorbed, facilitating the plant’s nutritional balance [31,32]. Moreover, it has been shown that the application of nano-iron oxide increases the absorption and uptake of important macronutrients in several plant species [32,33,34]. On the other hand, the addition of other NPs such as Au and Ag at different concentrations positively affected in vitro growth and development of another *Lavandula* species, *L. angustifolia* [35].

It has been observed that NPs induced chloroplast disorganization, namely reducing the numbers of thylakoids and grana, resulting in lower chlorophyll amounts [24,36,37]. In agreement with these observations, most NPs treatments tested in this study caused a decrease in total chlorophyll and carotenoid contents in shoots of both species (Table 1). In the case of CuO NPs, this negative effect was strongly accentuated with the increase of NPs concentration (Table 1). El-Mahdy and Elazab [25] reported that the effect of ZnO NPs on photosynthetic pigments contents of shoots of two pomegranate cultivars strongly depended on NPs concentration. NPs significantly increased photosynthetic pigments contents at 1 and 2.5 mg/L, but reduced those contents at 7 and 10 mg/L. 

Under stress conditions, free radical production is stimulated which can cause an overproduction of MDA, a major peroxidation product of polyunsaturated fatty acids, and thus, MDA levels are frequently used as markers of oxidative damage. In this study, CuO NPs at 5 and 10 mg/L (29.24 and 31.78 nmol/g_fresh weight_), and Fe_3_O_4_ NPs at all concentrations tested (39.86, 36.57 and 41.23 nmol/g_fresh weight_) significantly increased MDA accumulation in comparison with the control in *L. viridis* (20.84 nmol/g_fresh weight_). On the other hand, in *T. lotocephalus* increases in MDA levels were observed with CuO NPs at 5 mg/L (29.2 nmol/g_fresh weight_), ZnO NPs at all concentrations (40.07, 43.42 and 36.17 nmol/g_fresh weight_, at 1, 5 and 10 mg/L, respectively), and Fe_3_O_4_ NPs at 1 mg/L (32.92 nmol/g_fresh weight_) compared with the control (23.92 nmol/g_fresh weight_). The increase of lipid peroxidation in response to CuO, ZnO and Fe_3_O_4_ NPs has been observed in several species [24,28,38,39]. According to Da Costa and Sharma [28] cell membrane is even the first target of CuO NPs toxicity. Chang et al. [39] reported that CuO-NPs can cause genotoxicity in plant tissues if the Cu amounts exceed the physiological tolerance range because Cu is indispensable to maintain homeostasis in living systems. Additionally, Zn is another essential microelement for plant growth and development, it stimulates protein synthesis and chlorophyll biosynthesis, and plays an important role in regulatory and physiological processes [25]. However, at high concentrations, Zn can induce lipid peroxidation as well as the production of H_2_O_2_ leading to the reduction of photosynthetic pigments biosynthesis [40]. 

### 2.2. Phenolic Profile Analyzed by HPLC-HR-MS

Phenolic compounds emerge as one of the main groups of plant secondary metabolites. They are linked with biotic and abiotic stress responses in plants and are the main responsible for the biological properties of plants, namely antioxidant activity. In addition to other well-known biotic and abiotic elicitors, NPs recently emerged as a new class of elicitors that can activate enzymatic pathways responsible for the production of secondary metabolites. Nevertheless, attempts to evaluate the impact of these submicron size particles on phenolics production are still limited. In this study, the phenolic profiles of *L. viridis* and *T. lotocephalus* extracts from shoots cultured in media with different NPs treatments were analyzed for the first time by HPLC-HR-MS. A total of thirteen (eleven phenolic acids, a flavonoid and a coumarin derivative) and twelve (ten phenolic acids, a flavonoid and a coumarin derivative) compounds were identified in *L. viridis* and *T. lotocephalus* extracts, respectively (Tables S1 and S2 and Table 2). Caffeic acid hexosides (I and II), fertaric acid, salvianolic acid derivatives, and herniarin were identified for the first time in *L. viridis* and *O*-caffeoylquinic acid, caffeic acid hexosides, salvianolic acid derivatives, luteolin-7-*O*-glucuronide and herniarin in *T. lotocephalus*. Apigenin and luteolin-7-*O*-glucuronide were the only flavonoid compounds detected in extracts from *L. viridis* and *T. lotocephalus*, respectively. The flavonoid luteolin was previously identified in *L. viridis* [12,41] and *T. lotocepahalus* [12] extracts but was not found in this study. The coumarin derivative herniarin was also found in all the extracts from both species (Table 2).

The hydroxycinnamic acid rosmarinic acid was outstandingly the major compound identified in the extracts of *L. viridis* (between 9450 mg/kg in 5 mg/L ZnO and 64,501 mg/kg in 10 mg/L CuO) and *T. lotocephalus* (between 10,413 mg/kg in 1 mg/L CuO and 65,998 mg/kg in 10 mg/L Fe_3_O_4_) (Table 2) as reported in previous studies with these species [8,11,12,14]. Rosmarinic acid contents in control extracts from *L. viridis* were significantly higher (59,874 mg/kg) than the values previously obtained by Costa et al. [12] (471.20, 41,293 and 32,046 mg/kg in water, water/ethanol and ethanol extract, respectively). In *T. lotocephalus*, rosmarinic acid content was higher (30,326 mg/kg) than the values obtained by Costa et al. [11] (23,100 and 22,600 mg/kg in water/ethanol and ethanol extract, respectively) but lower than the recently observed by Gonçalves et al. [14] (48,610 mg/kg in methanol extract).

The second most abundant compounds found in the extracts were salvianolic acid B in *L. viridis* (between 1181 mg/kg in 5 mg/L ZnO and 11,035 mg/kg in 1 mg/L CuO) and salvianolic acid A isomer I in *T. lotocephalus* (between 203 mg/kg in 1 mg/L ZnO and 1387 mg/kg in 5 mg/L CuO). Although being identified for the first time in these species, salvianolic acids were found in other *Lavandula* [42,43] and *Thymus* [44,45,46] species. Studies from Taghouti et al. [44] with several endemic Iberian *Thymus* species highlight the importance of salvianolic acids levels in *Thymus* extracts and their bioactivity.

In which concerns the effect of NPs on phenolics production, as observed for biometric features, the results depend on the species and NPs treatment. Although in *L. viridis* rosmarinic acid content was significantly enhanced by CuO NPs at 10 mg/L and salvianolic acid B by the same NPs at 1 and 5 mg/L, overall, total phenolic compounds were negatively affected by NPs, especially using ZnO and Fe_3_O_4_ NPs. On the other hand, NPs treatments including ZnO at 5 and 10 mg/L and Fe_3_O_4_ at all concentrations, significantly increased total phenolic compounds in *T. lotocephalus* as well as rosmarinic acid amounts. The effect of metal NPs on phenolics production is variable. ZnO NPs also induced an increase in total phenolic and flavonoid contents in callus of *Stevia rebaudiana*, but determined by spectrophotometric assays [47] and in cell suspension cultures from *Brassica nigra* seedlings [48]. ZnO NPs stimulated the production of thymol and carvacrol in callus cultures of *Thymus* species and *Zataria multiflora* [49]. Additionally, Fe_3_O_4_ NPs increased total phenolic, flavonoid, anthocyanin, and lignin contents in cell suspension cultures of *Dracocephalum polychaetum* and also, the contents of several phenolics like naringin, rutin, quercetin, apigenin, rosmarinic acid, thymol and carvacrol [50]. In *T. lotocephalus* caffeic acid hexoside was only found in quantifiable amounts in samples of shoots cultured in medium with Fe_3_O_4_ NPs at 10 mg/L. The highest contents of salvianolic acids in this species were obtained in extracts from shoots cultured in a medium with 5 mg/L of CuO NPs (Table 2). Abdel-Wahab et al. [38] also reported increases in phenolic contents cultured with CuO NPs in callus cultures of *Solanum nigrum*.

### 2.3. Antioxidant and Enzymes Inhibitory Activities: Correlation with Phenolic Composition

A plant extract is a complex mixture of several compounds with different abilities to terminate radical chain processes and, thus, its antioxidant capacity must be assessed by more than one assay. In this study the antioxidant activity was assessed by four chemical assays: one HAT-based method [oxygen radical absorbance capacity (ORAC)] and three mixed-mode (ET- and HAT-based) methods [2,2′-Azino-bis (3-ethylbenzothiazoline-6-sulfonic acid) (ABTS), 2,2-diphenyl-1-picrylhydrazyl (DPPH) and ferric reducing antioxidant power (FRAP)]. The ORAC assay measures the radical chain breaking capacity of antioxidants by determining the blocking-up of peroxyl radicals generated oxidation. ABTS and DPPH measure the scavenging ability toward stable free radicals and FRAP assay measures the ability of the extracts to reduce Fe^3+^ to Fe^2+^. The antioxidant activity measured by ABTS (185 ± 1 and 101.3 ± 0.6 mg_TE_/g_extract_, respectively for *L. viridis* and *T. lotocephalus* extracts), DPPH (167 ± 7 and 86 ± 2 mg_TE_/g_extract_), FRAP (229 ± 3 and 125 ± 1 mg_AAE_/g_extract_) and ORAC (619 ± 31 and 358 ± 35 mg_TE_/g_extract_) assays of extracts from shoots cultured in the control medium was greater in *L. viridis* than in *T. lotocephalus*. The antioxidant activity values previously obtained [11,12] with ABTS and ORAC assays of water/ethanol extracts from in vitro cultured shoots of *L. viridis* (213.78 ± 3.06 and 710.28 ± 40.54 mg_TE_/g_extract_, respectively) and *T. lotocephalus* (140.91 ± 3.90 and 605.70 ± 69.08 mg_TE_/g_extract_) were slightly higher than those of the methanol extracts investigated in this work. Gonçalves et al. [14] also evaluated the antioxidant activity of a methanol extract from *T. lotocephalus* shoots with ABTS (75.51 ± 0.89 mg_TE_/g_extract_), DPPH (105.98 ± 8.37 mg_TE_/g_extract_), FRAP (101.18 ± 4.33 mg_AAE_/g_extract_) and ORAC (454.36 ± 16.54 mg_TE_/g_extract_) assays and the results are in the same range as those obtained in this work.

**Figure 1:**
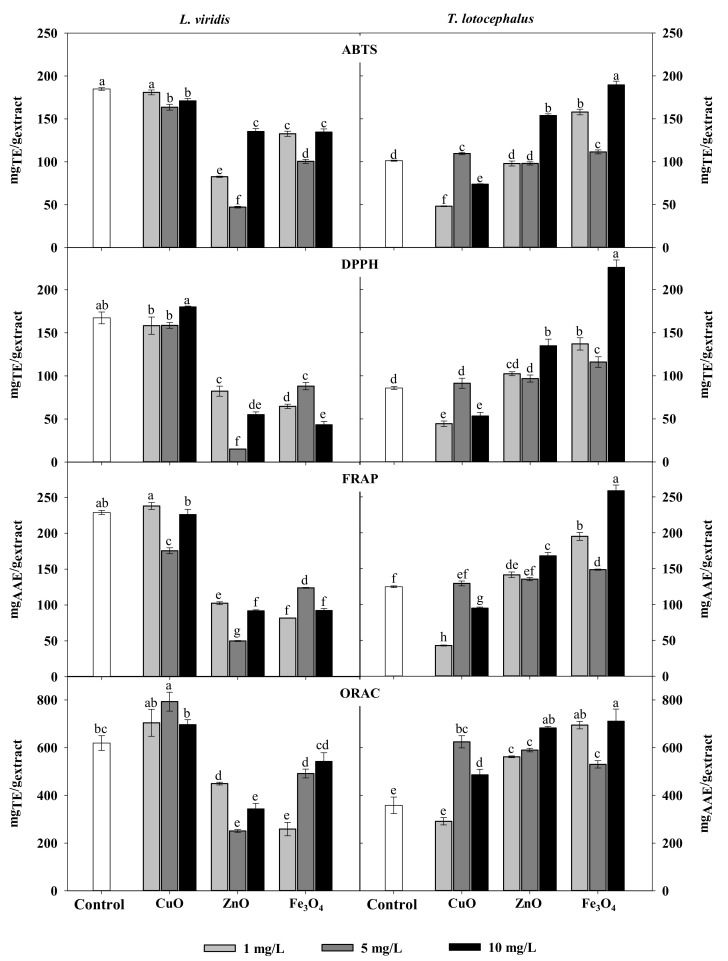
Antioxidant activity determined by ABTS, DPPH, FRAP, and ORAC assays of extracts from *Lavandula viridis* L’Hér and *Thymus lotocephalus* G. López and R. Morales shoots cultured in media with 0 (control), 1, 5 or 10 mg/mL of CuO, ZnO and Fe_3_O_4_ nanoparticles. Values are expressed as mean ± SE (*n* = 3). In each graph bars followed by different letters (a–f) are significantly different at *p* < 0.05 (Duncan’s New Multiple Range Test). ABTS: 2,2′-azino-bis(3-ethylbenzothiazoline-6-sulfonic acid); DPPH: 2,2-diphenyl-1-picrylhydrazyl; FRAP: ferric reducing antioxidant power; ORAC: oxygen radical absorbance capacity.

Regarding the impact of NPs on the antioxidant activity, it was observed that NPs treatments, particularly of ZnO and Fe_3_O_4_, lead to the decrease of antioxidant activity of *L. viridis* extracts probably as a result of the reduction in phenolic compounds (Table 2). On the other hand, ZnO, CuO and Fe_3_O_4_ NPs at some concentrations induced an increase in *T. lotocephalus* antioxidant activity (Figure 1). As observed for total phenolic contents, the highest free radical scavenging activity (ABTS^•+^ and DPPH^•^), as well as ferric reducing and peroxyl radical’s neutralization capacities of *T. lotocephalus* extracts, were obtained from shoots cultured with 10 mg/L of Fe_3_O_4_ NPs (189 ± 34 mg_TE_/g_extract_, 226 ± 9 mg_TE_/g_extract_, 259 ± 8 mg_AAE_/g_extract_ and 711 ± 51 mg_TE_/g_extract_, respectively). The greatest impact of NPs on the antioxidant activity in this species was measured with ORAC assay (Figure 1). This can be related to the different mode of action of this HAT-based assay that uses a fluorescent probe to compete with antioxidants for peroxyl radicals generated by the decomposition of 2,2′-azobis(2-methylpropionamidine) dihydrochloride (AAPH).

In this work CuO NPs at the concentrations tested did not improve the DPPH radical scavenging activity in *L. virids* and *T. lotocephalus*. Ahmad et al. [51] observed the highest DDPH scavenging activity of *Stevia rebaudiana* regenerants cultured with these NPs at 2 mg/L. Additionally, CuO and ZnO NPs also caused a significant increase in total antioxidant capacity, reducing power and DPPH radical scavenging capacity of the extracts from shoots of the same species [29,52]. There are no studies about the impact of the same type of NPs on the antioxidant activity of plants from the genus *Lavandula* and *Thymus* however, it was reported that Ag and Au NPs increased the ABTS free radical scavenging capacity in cultures of *L. angustifolia* [35].

A strong correlation was observed between antioxidant results obtained in the four assays and total phenolic contents of extracts from both species (*p* < 0.01) (Table 3) indicating that these compounds are the main ones involved in the antioxidant effects of these species. Moreover, in *L. viridis* a high correlation was also achieved between the contents of most of the individual phenolic compounds found in the extracts and the antioxidant results. In *T. lotocephalus* this correlation was observed for caffeic acid, rosmarinic acid, salvianolic acid A isomer III, luteolin-7-*O*-glucuronide and herniarin (Table 3). The correlation between phenolic compounds and antioxidant activity has been frequently reported for many species from *Thymus* and *Lavandula* genus [14,43,44]. Phenolics are plant secondary metabolites generally associated with plant defense mechanisms. They can act as antioxidants to scavenge ROS and thus, increase the phenolics production can be a strategy developed by plants to cope with stress-induced by NPs. Many biotic and abiotic stress factors can lead to oxidative stress induction in plants and stimulate the metabolism and accumulation of phenolic compounds [21]. Additionally, some studies indicated that the oxidative stress induced by the metal NPs in plants can influence the production of secondary metabolites, but this is still poorly understood [53,54].

Plants have been considered an important source of compounds with the capacity to inhibit enzymes linked to several pathologies. In this study the inhibitory effects of plant extracts to modulate key enzymes involved in neurodegenerative disorders (tyrosinase, acetylcholinesterase, and butyrylcholinesterase) and melanogenesis (tyrosinase) were investigated. The results are summarized in Table 4. As far as our literature review can ascertain this is the first report about the effect of the NPs addition to the culture medium on the enzyme inhibitory capacity of plant extracts.

The extracts from both species showed a similar capacity to inhibit tyrosinase activity; from 10.31 to 17.49 mg_KAE_/g_extract_ in *L. viridis* and 11.46 to 18.36 in mg_KAE_/g_extract_ in *T. lotocephalus*. Water extracts from *L. viridis* showed no inhibition of tyrosinase activity [55] and there are no previous reports describing the tyrosinase inhibitory capacity of *T. lotocephalus* extracts. CuO NPs at 5 and 10 mg/L significantly improved the anti-tyrosinase activity in *L. viridis*. On the other hand, in *T. lotocephalus* this activity increased with ZnO NPs at 10 mg/L, and Fe_3_O_4_ at 1 and 10 mg/L (Table 4).

A strong correlation was observed between the tyrosinase inhibitory activity of the extracts from both species and the total phenolic contents which is reported by other authors [56]. Additionally, this activity was strongly correlated with the amounts of some phenolic compounds including the major compound found in both species, rosmarinic acid. Indeed, this compound has been considered a good inhibitor of this enzyme [57,58]. The flavone luteolin-7-*O*-glucuronide identified in *T. lotocephalus* extracts appears to contribute also to the tyrosinase inhibitory effect of these extracts with a strong correlation. Recently, Mansinhos et al. [43] also reported a strong correlation between the contents of the flavone luteolin-7-*O*-glucoside and tyrosinase inhibition capacity of *Lavandula pedunculata* extracts.

The inhibitory capacities of the extracts against AChE and BChE ranged from 3.29–4.58 mg_GE_/g_extract_ and 2.49–12.33 mg_KAE_/g_extract_, for *L. viridis*, and from 2.86–3.74 mg_GE_/g_extract_ and 3.38–8.75 mg_GE_/g_extract_ for *T. lotocephalus*. Overall, NPs treatments showed no positive effect on anti-AChE activity of extracts from both species and on anti-BChE activity of extracts from *L. viridis*. Otherwise, all NPs at the highest concentration significantly enhanced the capacity of *T. lotocephalus* extracts to inhibit BChE enzyme.

The AChE inhibition capacity of the extracts was moderately correlated with total phenolic contents in *L. viridis* and no correlation was found in the case of *T. lotocephalus*. In which concerns the correlation of anti-cholinesterase activities and the contents of rosmarinic acid, the major compound in the extracts of both species, the results varied depending on the species. It was found a correlation in the case of *L. viridis*, that is moderate for AChE (*p* < 0.05) and strong for BChE (*p* < 0.01), and no correction in the case of *T. lotocephalus* extracts. Curiously, in this species, a strong correlation was observed with the contents of several salvianolic acid derivatives and the inhibitory activity against BChE (Table 3).

### 2.4. Principal Component Analysis (PCA)

**Figure 2:**
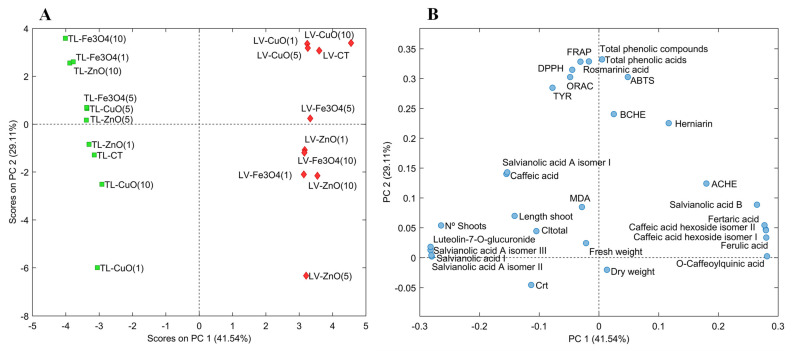
Score plot (**A**) and loading plot (**B**) of principal component analysis (PCA) of extracts from *Lavandula viridis* L’Hér (LV) and *Thymus lotocephalus* G. López and R. Morales (TL) shoots cultured in media with 0 (control), 1, 5 or 10 mg/mL of CuO, ZnO and Fe_3_O_4_ nanoparticles.

In the current study, PCA was employed to examine the effect of ZnO, CuO and Fe_3_O_4_ NPs at different concentrations (1–10 mg/L) on in vitro culture of the two Lamiaceae species *L. viridis* and *T. lotocephalus*, including their impact on plant growth (shoot number, length of the longest shoot and fresh and dry weight of the biomass produced), photosynthetic pigments production (total chlorophyll and carotenoid contents), lipid peroxidation (MDA content), chemical composition (individual phenolic contents) and biological properties [antioxidant (ORAC, DPPH, FRAP and ABTS) and enzyme (AChE, BChE and tyrosinase) inhibitory activities]. The score plot (Figure 2A) represents extracts from *L. viridis* (red rhombuses) and *T. lotocephalus* (green squares) with the different types and concentrations of NPs, and the loading plot (Figure 2B) represents the contribution of each studied parameter to the score. The first two principal components (PC) accounted for 70.65% of the total variation in the dataset, 41.54% and 29.11% for the first and second respectively. In the score plot, the PC1 showed a clear grouping of the extracts by plant species, while the PC2 separated the samples by the type and concentration of the NP tested. In general, it should be noted that the *T. lotocephalus* extracts obtained from shoots cultured on medium with Fe_3_O_4_ at the highest and lowest concentrations, as well as with ZnO at the highest concentration, contained the highest amount of phenolics (total phenolics, phenolic acids and rosmarinic acid) and presented the highest antioxidant activities (by FRAP, DPPH and ORAC assays), as well as the greater inhibition of the tyrosinase enzyme. These samples represented the highest contribution in the negative PC1 and positive PC2 with elevated contents of hydroxycinnamic acids, salvianolic acids A (isomer II and III) and I, and the flavone luteolin-7-*O*-glucuronide, compounds that also characterize the *T. lotocephalus* extracts from shoots cultured with Fe_3_O_4_, CuO and ZnO NPs all at 5 mg/L.

In the case of *L. viridis* extracts, a similar behavior was obtained with CuO NPs regardless of the concentration used. Furthermore, in general, extracts from these NPs presented the greatest response to the parameters studied and were similar to the control extract. The concentration of 10 mg/L of CuO improved the production of total phenolics (acids), and the contents of salvianolic acid B, O-caffeoylquinic, fertaric and ferulic acids, and the caffeic acid hexoside (isomers I and II). However, in the case of the hydroxycinnamic acids, these compounds were also in high contents when the shoots were cultured with Fe_3_O_4_ NPs at 5 mg/L. Furthermore, CuO and control *L. viridis* extracts presented higher antioxidant activity by ABTS and similar high activities for the other tested assays, and higher cholinesterase (BChE and AChE) inhibition than *T. lotocephalus* extracts.

## 3. Materials and Methods

### 3.1. Chemicals and Reagents

2,2′-Azino-bis (3-ethylbenzothiazoline-6-sulfonic acid) diammonium salt (ABTS) tablets, potassium persulfate (K_2_S_2_O_8_), DPPH, 6-benzyladenine (BA), methanol, trichloroacetic acid (TCA), 2-thiobarbituric acid (TBA), 5,5′-dithiobis(2-nitrobenzoic acid) (DTNB), 3,4-dihydroxy-l-phenylalanine (L-DOPA), acetylthiocholine iodide, S-butyrylthiocholine iodide, galantamine, AChE from Electrophorus electricus (electric eel, EC 3.1.1.7, Type VIS), BChE from equine serum horse-serum (EC 3.1.1.8), tyrosinase (EC 1.14.18.1) from mushroom, kojic acid, CuO nanopowder (<50 nm particle size), ZnO dispersion NPs (<100 nm particle size, ≤40 nm average particle size), and Fe_3_O_4_ nanopowder (50–100 nm particle size) were purchased from Sigma-Aldrich (Steinheim, Germany). (±)-6-Hydroxy-2,5,7,8-tetramethylchromane-2-carboxylic acid (Trolox), AAPH and fluorescein were supplied from Acros Organics (Geel, Belgium). Ascorbic acid was acquired from Merck (Darmstadt, Germany). Pure acetone was purchased from José Manuel Gomes dos Santos, Lda (Odivelas, Portugal) and Agar from Duchefa Biochemie B.V. (Haarlem, The Netherlands). Ferulic acid, caffeic acid and apigenin were obtained from AASC Ltd. (Southhampton, UK), and rosmarinic acid was acquired from Extrasynthese (Genay, France).

### 3.2. Plant Material, Treatments and Culture Conditions

In vitro shoot-cultures of *L. viridis* and *T. lotocephalus* multiplied in half strength MS medium (Murashige and Skoog 1962) with 0.2 mg/L 6-benzyladenine (BA) [22] and in MS medium [23], respectively, were used as plant material in the assays.

To evaluate the effect of NPs, 1, 5 or 10 mg/L of commercial CuO, ZnO or Fe_3_O_4_ NPs were added to the mentioned multiplication medium of each species. NPs were dissolved directly in distilled water, dispersed by an ultrasonic bath (Elma Hans Schmidbauer GmbH and Co. KG, Singen, Germany) for 40 min, and added at an appropriate amount to the culture medium before autoclaving. Medium with no NPs was used as control treatment. All media contained 2% (*w*/*v*) sucrose and 0.7% (*w*/*v*) agar and were autoclaved at 121 °C for 20 min. Shoots were cultivated in Erlenmeyer flasks containing 80 mL of agar-solidified medium. Cultures were incubated at 25 ± 2 °C with a 16-h photoperiod (cool white fluorescent lamps, 40 µmol m^−2^ s^−1^) and subcultured every 6 weeks. For each treatment 12 Erlenmeyer flasks with 7 shoots each were tested.

### 3.3. Biometric Features

After 6 weeks of treatment, the total number of shoots produced per culture, the length of the longest shoot and the fresh and dry weight of the biomass produced were recorded. Fresh and dry weight measurements were determined in all shoots produced per Erlenmeyer flask. Plant material was oven-dried (at 63 °C) until they reached a constant weight for the determination of dry weight.

### 3.4. Photosynthetic Pigments Analysis

Photosynthetic pigments were extracted from fresh plant material (25 mg) using 100% acetone (4 mL). The absorbance of the extract solutions was measured in a spectrophotometer at 661.6, 644.8 and 470 nm. Pigment contents were estimated as described by Lichtenthaler [59] using the equatuons below:

Cla (µg/mL) = 11.24 × Abs_661.6_ − 2.04 × Abs_644.8_

Clb (µg/mL) = 20.13 × Abs_644.8_ − 4.19 × Abs_661.6_

Cltotal (µg/mL) = 7.05 × Abs_661.6_ + 18.09 × Abs_644.8_

Crt (µg/mL) = (1000 × Abs_470_ − 1.90 × Cla − 63.14 × Clb)/214

### 3.5. Determination of Lipid Peroxidation

The quantification of malondialdehyde (MDA) content was used as an estimation of lipid peroxidation according to the method described by Hodges et al. [60]. Fresh plant material (100 mg) was crushed in a TCA solution (0.1 %, *w*/*v*) and centrifuged at 10,000× *g* for 5 min. The supernatant obtained was added to either 20 % (*w*/*v*) TCA (-TBA solution) or 0.5% (*w/v*) TBA in 20 % (*w/v*) TCA (+TBA solution). These mixtures were incubated at 95 °C for 30 min and then cooled in an ice bath. After centrifugation (3000× *g* for 10 min) the absorbance of the supernatant was measured at 532, 600 and 440 nm and the MDA equivalents were calculated using the equations below [60]:

Equivalents MDA (nmol/mL) = (A − B)/157,000 × 10^6^

A = (Abs_532+TBA_ − Abs_600+TBA_) − (Abs_532-TBA_ − Abs_600-TBA_)

B = (Abs_440+TBA_ − Abs_600+TBA_) × 0.0571

### 3.6. Extraction of Phenolics

The regenerated shoots were dried (40 °C) until constant weight and powdered (<2 mm particle size). The plant material was extracted with methanol (500 mg shoot material/30 mL solvent) for 30 min in an ultrasound water bath (Elma Hans Schmidbauer GmbH and Co. KG, Singen, Germany) at room temperature. The obtained extracts were filtered, the solvent was evaporated in a rotary evaporator under reduced pressure (40 °C), and stored at −20 °C until use.

### 3.7. HPLC-HR-MS Analysis of Phenolic Compounds

The phenolic compounds profile of plant extracts (1:100) was analysed using a Dionex Ultimate 3000 HPLC system comprising of a HPLC pump and an autosampler operating at 4 °C (Thermo Scientific, San Jose, CA, USA). Briefly, the separation of compounds was carried out with a 150 × 4.6 mm i.d. 5 μm 100 A C18 Kinetex column (Phenomenex, Macclesfield, UK). The injection volume was 5 μL and the separation was obtained at a flow rate of 1 mL/min maintained at 40 °C. The chromatographic conditions were carried out following those used by Mansinhos et al. [43]. After the PDA detector, part of the column eluate went (0.2 mL/min) directly to an Exactive Orbitrap mass spectrometer (Thermo Fisher Scientific, San José, CA, USA) fitted with a heated electrospray ionization probe (HESI) operating in negative ionization mode. Full scans were recorded in *m*/*z* range from 100 to 1000 with a resolution of 50,000 Hz and with a full automatic gain control (AGC) target of 1,000,000 charges, using 2 microscans. The analyses were also based on scans with in-source collision-induced dissociation (CID) at 25 eV. The MS experimental conditions with HESI in negative ionization mode were: capillary temperature of 320 °C, heater temperature of 150 °C, the sheath gas was 25 units, the auxiliary gas was 5 units, and the spray voltage was 4 kV.

Data acquisition and processing were carried out using Xcalibur software (Thermo Fisher Scientific, CA, USA). The Exactive Orbitrap was externally calibrated weekly using ready-to-use calibration mixtures (Pierce ESI Negative Ion Calibration Solution and Pierce LQT ESI Positive Ion Calibration Solution, both available from Thermo Fisher Scientific, San José, CA, USA). Quality control (QC) samples were applied to assess and verify the analytical process. The QC samples, composed of identical aliquots of a representative pool of the plant extracts, were injected regularly throughout the run. This QC sample represented both the sample matrix and metabolite composition of the samples and was used to monitor drifts and to determine the variance of a metabolite feature (below 20%).

Tentative identification of phenolic compounds (Table S1 was achieved as follows: (a) by comparing the exact mass and the retention time (RT) with the available standards and (b) in the absence of standards, compounds were tentatively identified by comparing the theoretical exact mass of the molecular ion with the measured accurate mass of the molecular ion and searched against Metlin, Phenol Explorer, PubChem and ChemSpider metabolite databases. In addition, these compounds were previously identified in plants of the same genus [8,14,44,61]. Metabolites having molecular masses within the pre-specified tolerance (mass difference less than 5 ppm) of the query masses are retrieved from these databases. Additionally, the identification of compounds was carried out following the MSI MS levels previously established by Sumner et al. [62], in which the metabolites identified using *m*/*z*, RT and/or MS/MS of reference standards were classified in level 1 and putatively annotated compounds using *m*/*z*, RT and/or MS/MS from the spectral library and no reference standards were labelled in level 2. The characteristics such as exact mass, delta ppm between experimental, retention time and MSI MI level are summarized in Supplementary Table S1. Phenolic compounds were quantified using the areas of their chromatographic peaks by selecting the theoretical exact mass of the molecular ion by reference to standard curves. In absence of reference compounds, they were quantified by reference to the calibration curve of a closely related parent compound (based on their structures). The linearity was determined for all the available standards. Limits of detection (LOD) and limits of quantification (LOQ) were estimated from the standard deviation of ten determinations of a blank. LOD and LOQ ranged from 0.00 to 0.50 mg/L and 0.01 to 1.68 mg/L, respectively. The different parameters used in the quantification of phenolic compounds are summarized in Supplementary Table S2. All the analyses were performed in triplicate.

### 3.8. Antioxidant Activity

The antioxidant activity results were expressed in equivalents of standard compounds calculated using the regression equations of standard plots obtained in each method.

#### 3.8.1. ABTS Free Radical Scavenging Assay

A stock solution of ABTS^•+^, prepared using potassium persulfate as the oxidizing agent, was diluted to give an absorbance of 0.700 at 734 nm [63]. The absorbance of 190 µL of this solution with 10 µL of the plant extract was measured at 734 nm. The extract dilution that produced 20–80% inhibition of the blank absorbance (inhibition (%) = (1 − Abs_extract_/A_blank_) × 100) was used to calculate the results that were expressed as milligrams of Trolox equivalents per gram of extract (mg_TE_/g_extract_). 

#### 3.8.2. DPPH Free Radical Scavenging Assay

This assay was performed according to the method described by Soler-Rivas et al. [64] with some modifications. A mixture containing 30 µL of plant extract, 300 µL of a 90 µM DPPH methanolic solution and 570 µL of methanol was incubated at room temperature for 30 min before reading the absorbance at 515 nm. The extract dilution that produced 20–80% inhibition of the blank absorbance was used to calculate the results that were expressed as milligrams of Trolox equivalents (TE) per gram of extract (mg_TE_/g_extract_).

#### 3.8.3. Ferric Reducing Antioxidant Power (FRAP)

The reducing properties of the extracts were determined according to the method described by Pulido et al. [65]. For that, a mixture containing 100 µL of plant extract, 250 µL of sodium phosphate buffer (200 mM, pH 6.6) and 250 µL of 1% K_3_[Fe(CN)_6_] was incubated at 50 °C (20 min). Then, 250 µL of 10% TCA were added to this mixture that was centrifuged. The obtained supernatant was mixed with the same amount of water (100 µL) and 20 µL of a FeCl_3_ solution (0.1%) and the absorbance was measured at 700 nm. The results were expressed as milligrams of ascorbic acid equivalents per gram of extract (mg_AAE_/g_extract_).

#### 3.8.4. Oxygen Radical Absorbance Capacity (ORAC) Assay

The ORAC assay was conducted using AAPH as a peroxyl radical generator [66]. A mixture containing 150 µL of 0.2 µM fluorescein solution (fluorescent probe) and 25 µL of plant extract was incubated at 37 °C in microplates. After 10 min of the incubation period, 25 µL of 150 mM AAPH solution were added to each well and the reduction in fluorescence was measured by reading fluorescein excitation at 485 nm and emission at 530 nm for 90 min. The ORAC values were calculated using the quadratic regression equation obtained from concentrations of Trolox stock solutions and the area under the curve (AUC). Results were expressed as milligrams of TE per gram of extract (mg_TE_/g_extract_).

### 3.9. Enzyme Inhibitory Capacity

#### 3.9.1. Tyrosinase

Tyrosinase activity was measured according to the method described by Masuda et al. [67]. Sodium phosphate buffer (20 mM, pH 6.8) (80 µL) was mixed with a 46 U/mL tyrosinase solution prepared in buffer (40 µL) and the plant extract (40 µL). After incubation at room temperature for 10 min 40 µL of l-3,4-dihydroxyphenylalanine (l-DOPA, substrate) were added and after another incubation period of 10 min the absorbance of the mixture was measured at 475 nm. A blank sample without enzyme was also performed to eliminate some interference of the extract colour. The inhibition percentages were calculated against a blank without extract and the results were expressed as milligrams of kojic acid equivalents per gram of extract calculated using the regression equation of a standard plot (mg_KAE_/g_extract_).

#### 3.9.2. Cholinesterases

The evaluation of AChE and BChE inhibitory activity was based on Ellman’s method [68], using a 96-well microplate reader. Initially, 125 µL of 3 mM DTNB solution, 25 µL of 15 mM substrate solution (acetylthiocholine iodide or S-butyrylthiocholine), 50 µL of 100 mM phosphate buffer (pH 8.0) and 25 µL of plant extract were mixed. Then, 25 µL of enzyme solution (0.28 U/mL) were added to the mixture and the absorbance was read at 405 nm. The inhibition percentages were calculated against a blank without extract and the results were expressed as milligrams of galantamine equivalents per gram of extract calculated using the regression equation of a standard plot (mg_GE_/g_extract_).

### 3.10. Statistical Analysis

The data were presented as the means ± standard errors of three replicates of each experiment. Data were analyzed by one-way analysis of variance (ANOVA), and Duncan’s New Multiple Range Test (*p* < 0.05), and correlations were calculated using Pearson’s test. Statistical analyses were carried out using IBM SPSS Statistics for Windows, Version 25.0. Armonk, NY: IBM Corp. Data were auto-scaled and a principal component analysis (PCA) was performed using the statistical software SOLO v. 8.6 (Eigenvector Research Inc., Manson, WA, USA).

## 4. Conclusions

Our results suggest that NPs added to culture media have a significant impact on different parameters of *L. viridis* and *T. lotocephalus* in vitro shoot cultures. The results varied with the species but also with the NP treatment. In *T. lotocephalus* ZnO and Fe_3_O_4_ have a positive effect on phenolics production and antioxidant activity suggesting that NPs can be useful as chemical elicitors in this species. However, in *L. viridis* only CuO NPs at 10 mg/L slightly improved the chemical contents and biological activities, suggesting that other types of NPs should be tested to induce a higher production of bioactive compounds in this species. Although this research opens new avenues for studying different nanoelicitors for the exploration of in vitro culture as a beneficial biotechnological strategy for the production of plant bioactivities, more detailed work is required using NPs adequately characterized. 

## Figures and Tables

**Table 1:** Shoot growth and total chlorophyll (Cltotal), carotenoids (Crt) and malondialdehyde (MDA) contents in *Lavandula viridis* L’Hér and *Thymus lotocephalus* G. López and R. Morales shoots cultured in media with 0 (control), 1, 5 or 10 mg/L of CuO, ZnO and Fe_3_O_4_ nanoparticles.

Treatment	NP Concentration (mg/L)	No. Shoots	Length of the Longest Shoot (mm)	Fresh Weight (mg)	Dry Weight (mg)	Cltotal (mg/g_fresh weight_)	Crt (mg/g_fresh weight_)	MDA (nmol/g_fresh weight_)
*L. viridis*								
Control	0	4.26 ± 0.23 ^a^	33.5 ± 1.10 ^a^	1277 ± 196 ^b^	167 ± 19 ^b^	1.40 ± 0.08 ^a^	0.30± 0.02 ^a^	20.8 ± 1.5 ^d^
CuO	1	3.46 ± 0.22 ^b,c^	28.3 ± 0.9 ^b^	1836 ± 257 ^a,b^	207 ± 19 ^a,b^	1.40 ± 0.08 ^a^	0.30 ± 0.01 ^a^	24.0 ± 1.2 ^d^
	5	2.90 ± 0.18 ^e,f^	24.4 ± 0.9 ^c^	2103 ± 451 ^a,b^	220 ± 29 ^a,b^	0.97 ± 0.02 ^b^	0.21 ± 0.01 ^b^	29.2 ± 1.6 ^c^
	10	2.84 ± 0.19 ^f^	21.4 ± 0.8 ^d^	1275 ± 152 ^b^	167 ± 16 ^b^	0.71 ± 0.05 ^c^	0.06 ± 0.01 ^e^	31.8 ± 2.4 ^b,c^
ZnO	1	3.07 ± 0.21 ^c,d^	24.6 ± 1.01 ^c^	2711 ± 364 ^a^	282 ± 27 ^a^	0.87 ± 0.07 ^b,c^	0.19 ± 0.01 ^b,c^	19.9 ± 0.8 ^d^
	5	2.68 ± 0.21 ^c,d,e^	28.7 ± 1.4 ^b^	2198 ± 366 ^a,b^	234 ± 32 ^a,b^	0.74 ± 0.05 ^b,c^	0.16 ± 0.01 ^c,d^	19.4 ± 1.6 ^d^
	10	2.33 ± 0.17 ^c,d,e^	20.6 ± 0.6 ^d^	2782 ± 330 ^a^	277 ± 25 ^a^	0.40 ± 0.05 ^d^	0.13 ± 0.01 ^d^	20.7 ± 1.0 ^d^
Fe_3_O_4_	1	4.41 ± 0.28 ^a^	27.5 ± 1.1 ^b,c^	2249 ± 356 ^a,b^	260 ± 29 ^a^	0.69 ± 0.04 ^c^	0.16 ±.0 01 ^c,d^	39.9 ± 2.2 ^a^
	5	4.24 ± 0.29 ^a^	26.1 ± 1.3 ^b,c^	2787 ± 425 ^a^	251 ± 31 ^a,b^	0.75 ± 0.08 ^b,c^	0.15 ± 0.01 ^c,d^	36.6 ± 2.4 ^a,b^
	10	3.92 ± 0.24 ^a,b^	29.3 ± 1.3 ^b^	2761 ± 394 ^a^	268 ± 31 ^a^	0.77 ± 0.06 ^b,c^	0.19 ± 0.01 ^b,c^	41.2 ± 2.0 ^a^
*T. lotocephalus*								
Control	0	16.28 ± 2.83 ^b,c^	38.30 ± 2.34 ^a^	1902.07 ± 443.18 ^b,c,d^	174.43 ± 29.21 ^b,c,d^	1.35 ± 0.05 ^b^	0.29 ± 0.02 ^b^	23.92 ± 1.86 ^e^
CuO	1	8.19 ± 1.02 ^d^	29.44 ± 1.55 ^b^	1397.72 ± 374.06 ^c,d^	214.41 ± 80.99 ^b,c,d^	1.57 ± 0.11 ^a^	0.34 ± 0.03 ^a^	21.76 ± 0.63 ^e^
	5	8.66 ± 1.67 ^d^	17.54 ± 0.81 ^c^	1211.88 ± 292.58 ^d^	138.55 ± 21.28 ^c,d^	1.24 ± 0.09 ^b,c^	0.27 ± 0.02 ^b,c^	29.20 ± 1.73 ^d^
	10	11.19 ± 1.78 ^c,d^	20.79 ± 1.11 ^c^	1086.10 ± 193.89 ^d^	132.59 ± 20.65 ^d^	1.03 ± 0.07 ^c,d,e^	0.23 ± 0.01 ^c,d,e^	20.55 ± 2.00 ^e^
ZnO	1	19.70 ± 2.35 ^b^	36.76 ± 2.11 ^a^	2254.67 ± 443.50 ^b,c,d^	252.42 ± 30.06 ^a,b,c^	0.81 ± 0.05 ^e,f^	0.22 ± 0.01 ^d,e^	40.07 ± 1.83 ^a,b^
	5	17.93 ± 2.12 ^b,c^	38.63 ± 2.03 ^a^	2597.08 ± 455.25 ^b,c^	255.58 ± 31.63 ^a,b,c^	1.10 ± 0.04 ^c,d^	0.26 ± 0.01 ^b,c,d^	43.42 ± 1.55 ^a^
	10	16.80 ± 2.04 ^b,c^	41.86 ± 2.57 ^a^	2661.83 ± 349.44 ^b,c^	261.49 ± 21.18 ^a,b^	0.94 ± 0.07 ^d,e,f^	0.18 ± 0.01 ^e,f^	36.17 ± 1.83 ^b,c^
Fe_3_O_4_	1	17.69 ± 1.76 ^b,c^	40.30 ± 2.02 ^a^	2366.82 ± 391.22 ^b,c,d^	220.85 ± 27.78 ^b,c,d^	1.13 ± 0.08 ^c,d^	0.22 ± 0.02 ^d,e^	32.92 ± 1.70 ^c,d^
	5	31.62 ± 2.98 ^a^	38.08 ± 1.93 ^a^	4854.29 ± 769.03 ^a^	343.31 ± 39.90 ^a^	0.72 ± 0.05 ^f^	0.14 ± 0.01 ^f^	23.67 ± 1.58 ^e^
	10	26.35 ± 3.85 ^a^	41.95 ± 1.73 ^a^	3116.72 ± 381.17 ^b^	271.61 ± 27.06 ^a,b^	0.83 ± 0.07 ^e,f^	0.18 ± 0.01 ^e,f^	23.21 ± 2.04 ^e^

Values are expressed as mean ± standard error. For each variable and plant species values followed by different letters (a to f) are significantly different at *p* < 0.05 (Duncan’s New Multiple Range Test).

**Table 2:** Concentration (mg/kg of extract, mean ± standard error) of phenolic compounds present in the extracts from *Lavandula viridis* L’Hér and *Thymus lotocephalus* G. López and R. Morales shoots cultured in media with 0 (control), 1, 5 or 10 mg/L of CuO, ZnO and Fe_3_O_4_ nanoparticles.

Compound	Treatment									
	Control	CuO (mg/L)			ZnO (mg/L)			Fe_3_O_4_ (mg/L)		
		1	5	10	1	5	10	1	5	10
*L. viridis*										
*O*-Caffeoylquinic acid	81 ± 8 ^a^	92 ± 7 ^a^	83 ± 0 ^a^	80.6 ± 0.7 ^a^	97 ± 7 ^a^	101 ± 2 ^a^	100 ± 5 ^a^	83 ± 10 ^a^	83 ± 3 ^a^	91 ± 8 ^a^
Caffeic acid hexoside isomer I	1039 ± 80 ^b^	1229 ± 85 ^a,b^	583 ± 32 ^c^	1400 ± 71 ^a^	171 ± 126 ^d^	144 ± 1 ^d^	221 ± 7 ^d^	221 ± 13 ^d^	325 ± 27 ^d^	231 ± 13 ^d^
Caffeic acid hexoside isomer II	159 ± 3 ^b^	1866 ± 16 ^b^	1091 ± 29 ^c^	2269 ± 170 ^a^	258 ± 207 ^d,e^	212 ± 7 ^e^	331.1 ± 0.5 ^d,e^	341 ± 35 ^d,e^	564 ± 96 ^d^	369 ± 55 ^d,e^
Fertaric acid	227 ± 16 ^a^	119 ± 5 ^b^	70 ± 3 ^c^	233 ± 9 ^a^	52 ± 2 ^c,d^	27.6 ± 0.4 ^e^	23.0 ± 0.1 ^e^	38 ± 1 ^d,e^	50 ± 4 ^c,d^	35 ± 1 ^d,e^
Caffeic acid	147.2 ± 0.5	<LOQ	<LOQ	<LOQ	<LOD	<LOD	<LOD	<LOD	<LOQ	<LOD
Ferulic acid	2996 ± 134 ^a^	2336 ± 173 ^b^	1328 ± 14 ^d^	1650 ± 29 ^c^	1247 ± 48 ^d^	291 ± 3 ^f^	499 ± 36 ^f^	827 ± 44 ^e^	1352 ± 8 ^d^	751 ± 16 ^e^
Rosmarinic acid	59874 ± 1040 ^b^	59183 ± 1429 ^b^	49922 ± 772 ^c^	64501 ± 2858 ^a^	27262 ± 47 ^e^	9450± 2 ^g^	20505 ± 250 ^f^	17086 ± 483 ^f^	32747 ± 1317 ^d^	19656 ± 587 ^f^
Salvianolic acid A isomer I	287 ± 24 ^c^	415 ± 24 ^b^	484 ± 23 ^a^	108 ± 1 ^e^	169 ± 6 ^d^	86 ± 2 ^e^	206 ± 8 ^d^	118.0 ± 0.4 ^e^	197 ± 4 ^d^	106 ± 3 ^e^
Salvianolic acid A isomer II	<LOQ	50 ± 8	52 ± 10	<LOQ	<LOQ	<LOD	<LOQ	<LOD	<LOQ	<LOD
Salvianolic acid I	<LOQ	49.0 ± 0.1	45.2 ± 0.2	<LOQ	<LOQ	<LOQ	<LOD	<LOQ	<LOQ	<LOD
Salvianolic acid B	7281 ± 534 ^c^	11035 ± 654 ^a^	9545 ± 449 ^b^	3673 ± 55 ^d^	3344 ± 63 ^d^	1181 ± 44 ^e^	1612 ± 34 ^e^	2093 ± 61 ^e^	4078 ± 2 ^d^	2072 ± 91 ^e^
*Total phenolic acids*	73522 ± 1818 ^a^	76374 ± 2371 ^a^	63203 ± 1253 ^b^	73916 ± 3196 ^a^	32599 ± 489 ^d^	11494 ± 51 ^f^	23498 ± 330 ^e^	20808 ± 651 ^e^	39396 ± 1433 ^c^	23311 ± 741 ^e^
Apigenin	<LOQ	<LOQ	<LOQ	63.78 ± 2.17	<LOD	<LOD	<LOD	<LOD	<LOQ	<LOD
Herniarin	519 ± 26 ^b^	733 ± 7 ^a^	521 ± 31 ^b^	142 ± 17 ^e^	259 ± 5 ^d^	71 ± 4 ^f^	238 ± 5 ^d^	147 ± 8 ^e^	361 ± 4 ^c^	177 ± 2 ^e^
*Total phenolic compounds*	74042 ± 1845 ^a^	77107 ± 2378 ^a^	63725 ± 1284^b^	74122 ± 3214 ^a^	32858 ± 494 ^d^	11565 ± 55 ^f^	23735 ± 325 ^e^	20955 ± 659 ^e^	39758 ± 1437 ^c^	23489 ± 739 ^e^
*T. lotocephalus*										
O-Caffeoylquinic acid	<LOQ	64 ± 5	<LOQ	<LOQ	<LOQ	<LOQ	n.d.	<LOQ	<LOQ	<LOQ
Caffeic acid hexoside isomer I	<LOQ	<LOQ	<LOQ	<LOQ	<LOQ	<LOQ	<LOQ	<LOQ	<LOQ	77 ± 3
Caffeic acid hexoside isomer II	<LOQ	<LOQ	<LOQ	<LOQ	<LOQ	<LOQ	<LOQ	<LOQ	<LOQ	74.2 ± 0.5
Caffeic acid	<LOQ	<LOD	91 ± 6 ^b^	<LOD	<LOQ	<LOQ	110 ± 9 ^b^	113 ± 3 ^b^	<LOQ	149 ± 3 ^a^
Rosmarinic acid	30326 ± 1453 ^e^	10413 ± 512 ^f^	31285 ± 3028 ^d,e^	15743 ± 1239 ^f^	31395 ± 697 ^d,e^	40635 ± 331 ^c,d^	64658 ± 6034 ^a^	55268 ± 5250 ^b^	46136 ± 851 ^b,c^	65998 ± 2478 ^a^
Salvianolic acid A isomer I	225 ± 5 ^e^	217 ± 8 ^e^	1387 ± 200 ^a^	755 ± 96 ^b,c^	203 ± 12 ^e^	207 ± 6 ^e^	234 ± 22 ^e^	561 ± 51 ^c,d^	434 ± 5 ^d,e^	891.2 ± 0.72 ^b^
Salvianolic acid A isomer II	57 ± 8 ^f,g^	55 ± 7 ^f,g^	303 ± 6 ^a^	158 ± 2 ^b^	56 ± 2 ^f,g^	50 ± 1 ^g^	70 ± 4 ^e,f^	96 ± 4 ^d^	75 ± 3 ^e^	123 ± 8 ^c^
Salvianolic acid I	116 ± 6 ^d^	113 ± 4 ^d^	666 ± 91 ^a^	339 ± 45 ^b^	129 ± 7 ^c,d^	119 ± 2 ^d^	182 ± 17 ^c,d^	238 ± 24 ^b,c^	178 ± 2 ^c,d^	294.6 ± 0.4 ^b^
Salvianolic acid B	53 ± 4 ^e^	45 ± 1 ^e^	240 ± 33 ^a^	146 ± 16 ^b,c^	52 ± 2 ^e^	77 ± 2 ^d,e^	69 ± 7 ^e^	132 ± 13 ^c^	111.7 ± 0.3 ^c,d^	184 ± 3 ^b^
Salvianolic acid A isomer III	78 ± 4 ^e,f^	45 ± 3 ^f^	156 ± 22 ^a,b^	75 ± 10 ^e,f^	91 ± 4 ^d,e^	120 ± 2 ^c,d^	182 ± 14 ^a^	170 ± 11 ^a,b^	77 ± 1 ^e,f^	144 ± 5 ^b,c^
*Total phenolic acids*	30855 ± 1481 ^e^	10952 ± 540 ^f^	34128 ± 3375 ^d,e^	17216 ± 1408 ^f^	31927 ± 719 ^d,e^	41208 ± 344 ^c,d^	65506 ± 6090 ^a,b^	56578 ± 5355 ^b^	47013 ± 839 ^c^	67936 ± 2461 ^a^
Luteolin-7-*O*-glucuronide	133 ± 2 ^d^	81 ± 4 ^e^	215 ± 21 ^b^	83 ± 8 ^e^	134 ± 6 ^d^	167 ± 6 ^c,d^	209 ± 23 ^b^	301 ± 20 ^a^	193.3 ± 0.6 ^b,c^	315 ± 3 ^a^
Herniarin	76 ± 6 ^e^	<LOQ	120 ± 16 ^c^	49 ± 4 ^f^	88 ± 2 ^d,e^	107 ± 3 ^c,d^	174 ± 3 ^b^	163 ± 9 ^b^	157 ± 3 ^b^	204 ± 12 ^a^
*Total phenolic compounds*	31064 ± 1488 ^e^	11033 ± 544 ^f^	34463 ± 3412 ^d,e^	17348 ± 1421 ^f^	32149 ± 724 ^d,e^	41483 ± 352 ^c,d^	65889 ± 6070 ^a,b^	57042 ± 5366 ^b^	47363 ± 842 ^c^	68456 ± 2469 ^a^

Notes: n.d.—not detected; LOD—limit of detection; LOQ—limit of quantification. The results were analysed using one-way analysis of variance (ANOVA) followed by Duncan’s New Multiple Range Test. Different letters (a to g) in each row and for each phenolic compound mean significant differences (*p* < 0.05) among treatments.

**Table 3:** Pearson’s correlation coefficients between antioxidant activity measured by the different assays (DPPH, FRAP, ABTS, and ORAC), enzyme inhibitory activities (AChE, BChE, and Tyr), total and individual phenolic compounds determined by HPLC-HR-MS.

Phenolic Compounds	Antioxidant Activity				Enzyme Inhibitory Activity		
	ABTS	DPPH	FRAP	ORAC	Tyr	AChE	BChE
*L. viridis*							
*O*-Caffeoylquinic acid	−0.492 *	−0.519 *	−0.443	−0.432	−0.517 *	−0.177	−0.542 *
Caffeic acid hexoside isomer I	0.778 **	0.879 **	0.941 **	0.766 **	0.440 *	0.475 *	0.897 **
Caffeic acid hexoside isomer II	0.783 **	0.903 **	0.942 **	0.788 **	0.501 *	0.515 *	0.910 **
Fertaric acid	0.674 **	0.824 **	0.853 **	0.631 **	0.284	0.605 **	0.785 **
Ferulic acid	0.702 **	0.841 **	0.881 **	0.712 **	0.402	0.392	0.733 **
Rosmarinic acid	0.811 **	0.975 **	0.985 **	0.876 **	0.632 **	0.508 *	0.927 **
Salvianolic acid A isomer I	0.576 **	0.613 **	0.591 **	0.669 **	0.655 **	−0.025	0.552 *
Salvianolic acid B	0.669 **	0.776 **	0.791 **	0.817 **	0.645 **	0.102	0.745 **
Herniarin	0.614 **	0.662 **	0.704 **	0.698 **	0.540 *	0.026	0.577 **
Total phenolic contents	0.815 **	0.973 **	0.988 **	0.890 **	0.642 **	0.462 *	0.926 **
*T. lotocephalus*							
Caffeic acid	0.910 **	0.970 **	0.932 **	0.032	0.310	−0.049	−0.203
Rosmarinic acid	0.928 **	0.873 **	0.891 **	0.794 **	0.888 **	−0.178	0.147
Salvianolic acid A isomer I	0.243	0.172	0.255	0.394	0.065	0.269	0.851 **
Salvianolic acid A isomer II	0.061	−0.032	0.032	0.306	−0.011	0.289	0.845 **
Salvianolic acid I	0.132	0.020	0.104	0.382	0.037	0.221	0.842 **
Salvianolic acid B	0.362	0.293	0.390	0.514 *	0.214	0.232	0.828 **
Salvianolic acid A isomer III	0.770 **	0.591 **	0.650 **	0.875 **	0.739 **	0.035	0.436
Luteolin-7-*O*-glucuronide	0.914 **	0.858 **	0.887 **	0.795 **	0.693 **	0.214	0.401
Herniarin	0.901 **	0.864 **	0.851 **	0.745 **	0.793 **	−0.055	0.289
Total phenolic contents	0.935 **	0.877 **	0.898 **	0.808 **	0.887 **	−0.165	0.183

DPPH: 2.2-diphenyl-1-picrylhydrazyl; FRAP: ferric reducing antioxidant power; ABTS: 2.2′-azino-bis(3-ethylbenzothiazoline-6-sulfonic acid); ORAC: oxygen radical absorbance capacity; Tyr: tyrosinase; AChE: acetylcholinesterase; BChE: butyrylcholinesterase. * Correlation is significant (*p* < 0.05). ** Correlation is significant (*p* < 0.01).

**Table 4:** Enzymes inhibitory capacity of extracts from *Lavandula viridis* L’Hér and *Thymus lotocephalus* López and Morales shoots cultured in media with 0 (control), 1, 5 or 10 mg/L of CuO, ZnO and Fe_3_O_4_ nanoparticles.

Treatment	NP Concentration (mg/L)	TYR (mg_KAE_/g_extract_)	AChE (mg_GE_/g_extract_)	BChE (mg_GE_/g_extract_)
*L. viridis*				
Control	0	13.27 ± 0.08 ^c^	4.43 ± 0.08 ^a^	9.74 ± 0.89 ^b^
CuO	1	14.90 ± 0.66 ^b,c^	3.44 ± 0.19 ^b^	10.71 ± 0.56 ^b^
	5	17.49 ± 0.36 ^a^	3.65 ± 0.37 ^b^	10.26 ± 0.26 ^b^
	10	15.87 ± 0.36 ^a,b^	4.58 ± 0.26 ^a^	12.33 ± 0.31 ^a^
ZnO	1	13.99 ± 0.47 ^c^	3.42 ± 0.15 ^b^	4.04 ± 0.21 ^d,e^
	5	10.31 ± 0.34 ^d^	3.30 ± 0.21 ^b^	2.49 ± 0.51 ^f^
	10	13.35 ± 0.31 ^c^	3.34 ± 0.23 ^b^	2.96 ± 0.44 ^e,f^
Fe_3_O_4_	1	13.64 ± 0.41 ^c^	3.29 ± 0.19 ^b^	5.12 ± 0.52 ^c,d^
	5	14.60 ± 0.66 ^b,c^	3.75 ± 0.19 ^b^	4.95 ± 0.23 ^c,d^
	10	13.39 ± 0.08 ^c^	3.63 ± 0.26 ^b^	6.13 ± 0.65 ^c^
*T. lotocephalus*				
Control	0	13.18 ± 0.58 ^d,e^	3.28 ± 0.19 ^a,b,c^	5.00 ± 0.20 ^d^
CuO	1	11.46 ± 0.67 ^e^	3.25 ± 0.28 ^a,b,c^	5.32 ± 0.32 ^c,d^
	5	14.51 ± 0.97 ^b,c,d^	3.44 ± 0.12 ^a,b^	8.75 ± 0.60 ^a^
	10	14.16 ± 0.31 ^c,d,e^	3.05 ± 0.12 ^b,c^	6.36 ± 0.28 ^c^
ZnO	1	13.88 ± 0.60 ^c,d,e^	2.86 ± 0.21 ^c^	3.38 ± 0.30 ^e^
	5	15.49 ± 0.70 ^a,b,c,d^	3.05 ± 0.11 ^b,c^	4.92 ± 0.29 ^d^
	10	18.36 ± 1.51 ^a^	2.90 ± 0.18 ^b,c^	6.51 ± 0.67 ^b,c^
Fe_3_O_4_	1	17.39 ± 0.74 ^a,b^	3.74 ± 0.13 ^a^	4.92 ± 0.61 ^d^
	5	15.68 ± 1.36 ^a,b,c,d^	2.90 ± 0.11 ^b,c^	5.27 ± 0.09 ^c,d^
	10	16.81 ± 1.04 ^a,b,c^	3.22 ± 0.12 ^a,b,c^	7.70 ± 0.32 ^b^

Values are expressed as mean ± standard error. For each variable and plant species values followed by different letters (a to e) are significantly different at *p* < 0.05 (Duncan’s New Multiple Range Test). TYR: tyrosinase; AChE: acetylcholinesterase; BChE: butyrylcholinesterase; KAE: kojic acid equivalents; GE: galantamine equivalents.

## Supplementary Materials

The following are available online. Table S1: HPLC-HR-MS data of identified phenolic compounds from *Lavandula viridis* L’Hér and *Thymus lotocephalus* G. Lopez and R. Morales extracts, Table S2: Summary of HPLC-HR-MS parameters for quantification of phenolic compounds in *Lavandula viridis* L’Hér and *Thymus lotocephalus* G. Lopez and R. Morales extracts.
