# Supplementary material for: Impact of Metallic Nanoparticles on In Vitro Culture, Phenolic Profile and Biological Activity of Two Mediterranean Lamiaceae Species: Lavandula viridis L’Hér and Thymus lotocephalus G. López and R. Morales
Source: Molecules. 2021 Oct 25;26(21):6427. doi: 10.3390/molecules26216427 (PMC8587770; doi:10.3390/molecules26216427)
Supplement: Supplementary file 1 [file molecules-26-06427-s001.zip › molecules-1357688-supplementary.pdf]

Supplementary information

Table S1. HPLC-HR-MS data of identified phenolic compounds from *Lavandula viridis* L'Hér and *Thymus lotocephalus* G. Lopez & R. Morales extracts.

Peak	Compound identity	Chemical formula	Theoretical exact mass $[M-H]^-$ *(m/z)*	Delta ppm (error)	RT (min)	MSI MI level*
1	O-Caffeoylquinic acid[a]	$C_{16}H_{18}O_9$	353.0867	2.103	1.48	2
2	Caffeic acid hexoside[a]	$C_{15}H_{18}O_9$	341.0867	-0.007	2.9	2
3	Caffeic acid hexoside[a]	$C_{15}H_{18}O_9$	341.0867	0.143	3.5	2
4	Fertaric acid[a]	$C_{14}H_{14}O_9$	325.0557	0.774	4.23	2
5	Caffeic acid[a]	$C_9H_8O_4$	179.0338	-0.604	4.59	1
6	Ferulic acid[a]	$C_{10}H_{10}O_4$	193.0495	-0.374	7.59	1
7	Luteolin-7-O-glucuronide[c]	$C_{21}H_{18}O_{12}$	461.0714	0.569	8.11	2
8	Rosmarinic acid[a]	$C_{18}H_{16}O_8$	359.0761	0.488	9.57	1
9	Salvianolic acid A isomer I[a]	$C_{26}H_{22}O_{10}$	493.1129	0.508	10.11	2
10	Salvianolic acid A isomer II[a]	$C_{26}H_{22}O_{10}$	493.1129	0.358	10.97	2
11	Salvianolic acid I[a]	$C_{27}H_{22}O_{12}$	537.1027	1.499	11.13	2
12	Salvianolic acid B[a]	$C_{36}H_{30}O_{16}$	717.1450	-0.049	11.23	2
13	Herniarin[b]	$C_{10}H_8O_3$	175.0389	-0.429	12.14	2
14	Salvianolic acid A isomer III[a]	$C_{26}H_{22}O_{10}$	493.1129	-0.132	12.8	2
15	Apigenin[c]	$C_{15}H_{10}O_5$	269.0444	0.172	14.8	1

Class of compounds: Phenolic acids ([a]Hydroxycinnamic acids); [b]Coumarin derivative; Flavonoids ([c]Flavones).

*Metabolite standards initiative metabolite identification (MSI MI) levels. Reference standards were available for all compounds identified at MSI MI level 1.

Table S2. Summary of HPLC-HR-MS parameters for quantification of phenolic compounds in *Lavandula viridis* L'Hér and *Thymus lotocephalus* G. Lopez & R. Morales extracts.

Compound	Linear range (mg/L)	Intercept	Slope	R^2	LOD (mg/mL)	LOQ (mg/mL)
Rosmarinic acid	0.1-100	-103410	1598747	0.9992	0.06	0.19
Caffeic acid	0.1-10	1436398	3950274	0.9990	0.50	1.68
Ferulic acid	0.2-60	2487	9717	0.9995	0.37	1.25
Gallic acid	0.2-15	-330994	2547719	0.9998	0.00	0.01
Chlorogenic acid	0.2-15	-243627	1363025	0.9993	0.00	0.01
Luteolin	0.2-15	1464030	5454860	0.9990	0.29	0.96
Apigenin	0.2-15	185082	6649959	0.9995	0.00	0.01
Naringenin	0.2-15	-314843	2143048	0.9996	0.37	1.23
p-Coumaric acid	0.2-15	-2024	73034	0.9999	0.22	0.72
